# Malignant peripheral nerve sheath tumor of the sigmoid colon: A case report and comparison with published cases

**DOI:** 10.1016/j.ijscr.2025.111799

**Published:** 2025-08-13

**Authors:** Mohammed Mohammed Al-Shehari, Khaled Sultan Galeb, Wael Abdu Ahmed, Mansour Al-hameli, Haitham Mohammed Jowah, Maria A. Qasem

**Affiliations:** a Department of Surgery, Faculty of Medicine and Health Science, Sana'a University, Sana'a City, Yemen; b Department of Surgery, Al-Thawra Modern General Hospital, Sana'a City, Yemen; c Department of Pathology, ILab Specialized Medical Laboratories, Sana'a City, Yemen; d Department of Radiology, Faculty of Medicine and Health Science, Sana'a University, Sana'a City, Yemen

**Keywords:** Malignant peripheral nerve sheath tumor, MPNST, Sigmoid colon, Case report, Immunohistochemistry, S-100, SOX-10, SCARE guidelines

## Abstract

**Introduction:**

Malignant peripheral nerve sheath tumors (MPNSTs) are rare and aggressive soft tissue sarcomas. Its occurrence in the gastrointestinal tract, particularly in the sigmoid colon, is exceptionally uncommon and poses significant diagnostic and therapeutic challenges.

**Case presentation:**

We report the case of a 46-year-old female who presented with a four-year history of intermittent rectal bleeding and lower abdominal pain. A diagnostic workup, including imaging and colonoscopy, revealed a large ulcerated polypoid mass at the rectosigmoid junction. The definitive diagnosis of MPNST was confirmed through histopathology and a comprehensive immunohistochemical panel, which demonstrated characteristic spindle cells positive for S-100 and SOX-10 and negative for markers of gastrointestinal stromal tumors (GIST) and smooth muscle neoplasms. The patient underwent successful complete surgical resection with clear margins, which resulted in favorable short-term outcomes.

**Discussion:**

This case highlights the diagnostic difficulties associated with colonic MPNSTs owing to their rarity and non-specific presentation. A comparison of eight published cases revealed variable outcomes, with recurrence rates approaching 40 %. This result underscores the indispensable role of immunohistochemistry in differentiating MPNST from other mesenchymal tumors. Complete surgical resection with clear margins remains the cornerstone for treatment.

**Conclusion:**

Continued long-term follow-up is essential for patients with colonic MPNST given the aggressive nature and high recurrence potential of this malignancy.

## Highlights

• Malignant peripheral nerve sheath tumors (MPNST) in the sigmoid colon are rare.
• Immunohistochemistry (S-100, SOX-10) is essential for a definitive diagnosis.
• The mainstay of treatment is complete surgical resection, with clear margins.
• Long-term surveillance is crucial owing to its high recurrence potential.

## Abbreviations

MPNST: Malignant Peripheral Nerve Sheath Tumor
GI: Gastrointestinal
GIST: Gastrointestinal Stromal Tumor
NF1: Neurofibromatosis Type 1
CT: Computed Tomography
MRI: Magnetic Resonance Imaging
CEA: Carcinoembryonic Antigen
IHC: Immunohistochemistry
SMA: Smooth Muscle Actin
H&E: Hematoxylin and Eosin
TSH: Thyroid-Stimulating Hormone
R0: Resection with no residual tumor cells (microscopically clear margins)

## 1 Introduction

Malignant peripheral nerve sheath tumors (MPNSTs) are rare and aggressive soft tissue sarcomas, comprising less than 5 % of all sarcomas [1]. Although up to 50 % of cases are associated with neurofibromatosis type 1 (NF1), they can also arise sporadically [2]. These tumors are characterized by aggressive biological behavior, with high rates of local recurrence and a propensity for distant metastasis [1].

Although MPNSTs typically arise from major peripheral nerves in the extremities and trunk, their occurrence within the gastrointestinal (GI) tract is exceptionally uncommon, posing significant diagnostic challenges [3]. Among GI locations, the colon was one of the least frequent sites. A comprehensive review of the published literature revealed fewer than ten documented cases of primary colonic MPNST, underscoring its extraordinary rarity [[4], [5], [6], [7], [8], [9], [10], [11], [12]]. This often contributes to diagnostic delays because the clinical and radiological features of colonic MPNSTs closely mimic those of more prevalent malignancies such as gastrointestinal stromal tumors (GISTs) and leiomyosarcomas [13].

This case report presents sporadic MPNST of the sigmoid colon in a 46-year-old female and provides a systematic comparison with previously published cases. Here, we highlight the diagnostic complexities, justify the treatment approach, and discuss the prognostic implications of this rare entity. This report was prepared in accordance with the SCARE 2025 guidelines [14].

## 2 Case presentation

A 46-year-old female presented with a four-year history of intermittent rectal bleeding and progressive lower abdominal pain **(**Table 1**)**. Bleeding was not associated with defecation and was accompanied by an 8-kg weight loss over the same period. Additional symptoms include dysphagia and occasional fever. She denied any history of trauma, chronic illnesses, or family history of cancer or genetic syndromes.

**Table 1:** Patient timeline.

Date	Clinical event	Key findings and actions taken
2020 – Nov 2024	Symptom Onset and Progression	Four-year history of intermittent per rectal bleeding, lower abdominal pain, and progressive weight loss (8 kg).
November 18, 2024	Initial Endoscopic Investigation	Rectosigmoidoscopy revealed a large, ulcerative polypoid mass at 22 cm from the anal verge. Multiple biopsies were obtained.
December 3, 2024	Radiological Staging	Abdominal/pelvic CT and MRI scans characterized a ~ 6 cm rectosigmoid soft tissue mass without evidence of distant metastasis.
December 22, 2024	Surgical Intervention	The patient underwent a lower midline laparotomy and sigmoid colectomy with primary anastomosis, achieving R0 resection.
January 12, 2025	Definitive Diagnosis	Final histopathology with a comprehensive immunohistochemical panel confirmed the diagnosis of high-grade Malignant Peripheral Nerve Sheath Tumor (MPNST).
June 2025	6-Month Post-operative Follow-up	The patient remained asymptomatic with no clinical or radiological evidence of local recurrence or distant disease.

Physical examination revealed a chronically ill, but hemodynamically stable patient with mild tenderness in the left lower quadrant. Importantly, thorough examination revealed no cutaneous stigmata of Neurofibromatosis Type 1 (NF1), including café-au-lait macules, axillary freckling, or cutaneous neurofibromas. Digital rectal examination results were unremarkable.

**Fig. 1:**
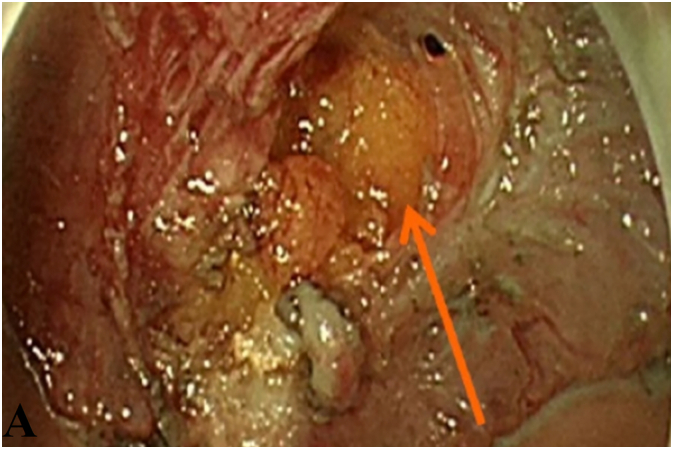
Endoscopic findings. Colonoscopy showing a large ulcerated polypoid mass (orange arrow) causing significant luminal narrowing.

On November 18, 2024, rectosigmoidoscopy revealed a large ulcerative polypoid mass, 22 cm from the anal verge, causing significant luminal narrowing (Fig. 1). Multiple biopsies were obtained for histopathological analysis. Initial laboratory investigations demonstrated anemia (hemoglobin 8.5 g/dL) and thrombocytosis (platelet count 730 × 10^9^/L), while carcinoembryonic antigen (CEA) levels remained within normal limits at 1.2 ng/mL (Table 2).

**Table 2:** Summary of laboratory and imaging investigations.

Investigation	Result	Normal Range
Hemoglobin	8.5 g/dL	12–15 g/dL
White blood cell count	4.99 × 10^9^/L	4–11 × 10^9^/L
Platelet count	730 × 10^9^/L	150–450 × 10^9^/L
Carcinoembryonic antigen	1.2 ng/mL	<5 ng/mL
TSH	3 uIU/mL	0.4–4.0 uIU/mL

**Fig. 2:**
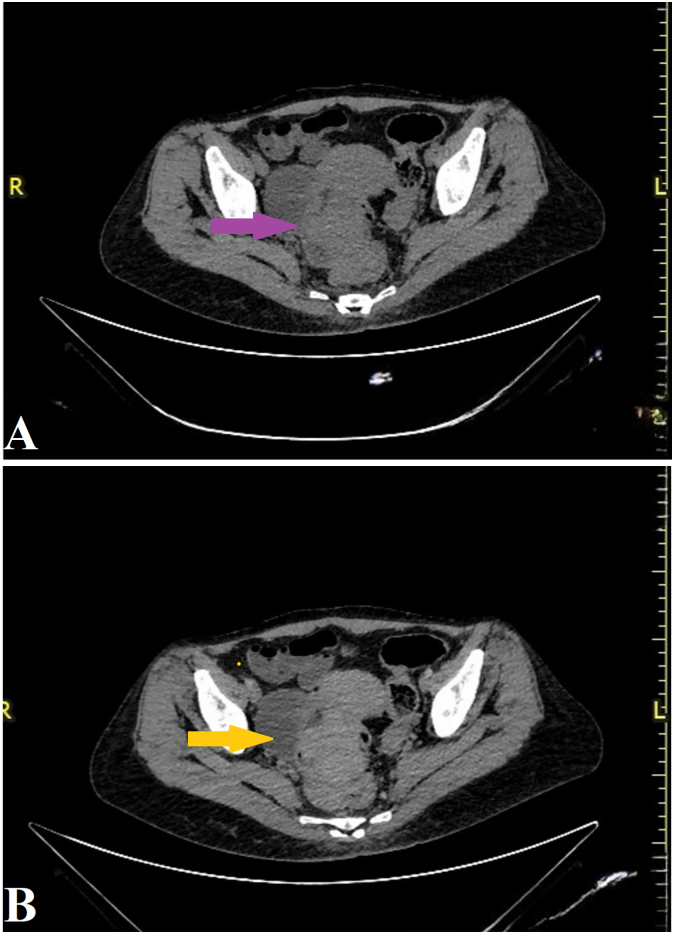
Abdominal computed tomography (CT) scan. (A) Axial non-contrast CT image showing a large, irregular polypoid mass (purple arrow) at the rectosigmoid junction. (B) Axial contrast-enhanced CT image demonstrating heterogeneous enhancement of the mass (golden arrow), measuring approximately 60 × 53 × 52 mm. (For interpretation of the references to colour in this figure legend, the reader is referred to the web version of this article.)

**Fig. 3:**
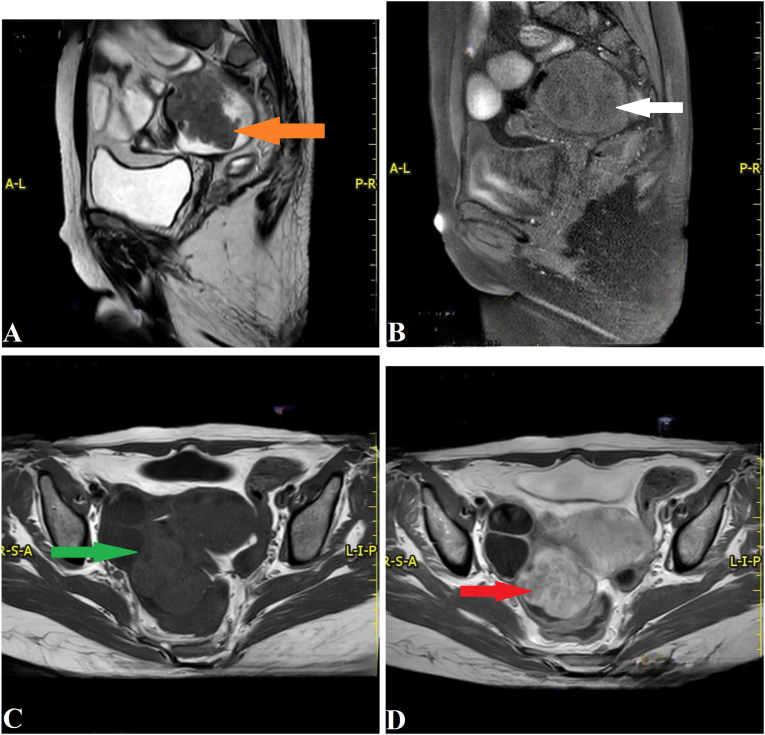
Pelvic magnetic resonance imaging (MRI). (A) Sagittal T2-weighted image showing a large polypoid mass with intermediate signal intensity (orange arrow). (B) Sagittal T1 fat-suppression pre-contrast image showing a mass with intermediate signal intensity (white arrow). (C) Axial T1-weighted image without contrast, demonstrating a mass with intermediate signal intensity (green arrow). (D) Axial T1-weighted post-contrast image showing avid enhancement of the tumor (red arrow). (For interpretation of the references to colour in this figure legend, the reader is referred to the web version of this article.)

Radiological staging performed on December 3, 2024, included abdominal computed tomography (CT) and pelvic magnetic resonance imaging (MRI). Computed tomography (CT) revealed a large irregular polypoid mass measuring 60 mm × 53 mm × 52 mm at the rectosigmoid junction with heterogeneous enhancement and adjacent mesenteric lymphadenopathy (Fig. 2). MRI further characterized the mass, demonstrating intermediate signal intensity on T1-weighted images and relatively high intermediate intensity on T2-weighted sequences, consistent with soft-tissue neoplasms (Fig. 3). Radiological differential diagnosis included gastrointestinal stromal tumor (GIST), leiomyosarcoma, adenocarcinoma, and other mesenchymal neoplasms. The heterogeneous enhancement pattern and intermediate signal characteristics on both T1 and T2- weighted sequences were more suggestive of a sarcomatous process than a typical adenocarcinoma. The absence of characteristic GIST enhancement patterns and a predominantly solid appearance helped narrow the differential diagnosis, although a definitive diagnosis required histopathological confirmation.

**Fig. 4:**
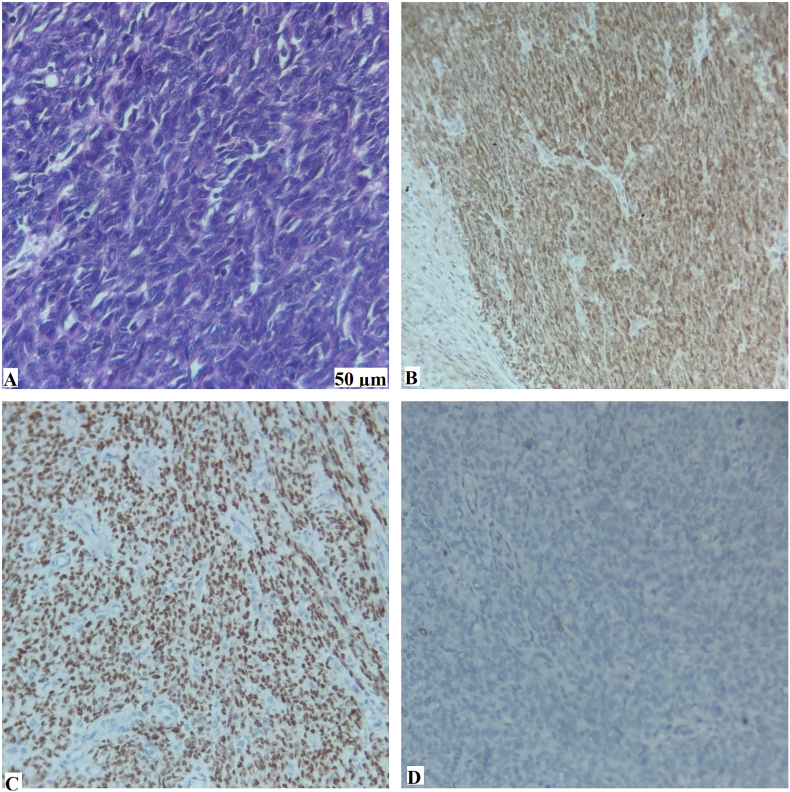
Histopathological, and immunohistochemical findings. (A) Hematoxylin and eosin (H&E) staining revealed an infiltrative tumor composed of spindle-shaped cells with nuclear pleomorphism. (B) Immunohistochemistry (IHC) showing diffuse positive staining for S-100. (C) IHC showing strong positive staining for SOX-10. (D) IHC showing negative staining for desmin, helping rule out a smooth muscle neoplasm.

Histopathological examination revealed an infiltrative tumor composed of spindle-shaped cells with marked nuclear pleomorphism and a high mitotic rate (5/10 high-power fields), suggestive of high-grade sarcoma (Fig. 4A). A comprehensive immunohistochemical (IHC) panel was used to establish a definitive diagnosis. The tumor cells demonstrated diffuse positivity for S-100 protein (Fig. 4B) and SOX-10 (Fig. 4C), confirming the origin of their neural crest. Conversely, negative staining for CD117, CD34, DOG-1, smooth muscle actin (SMA), and desmin (Fig. 4D) effectively excluded GIST and smooth muscle neoplasms. The Ki-67 proliferation index was 10 %, indicating high-risk malignant behavior.

**Fig. 5:**
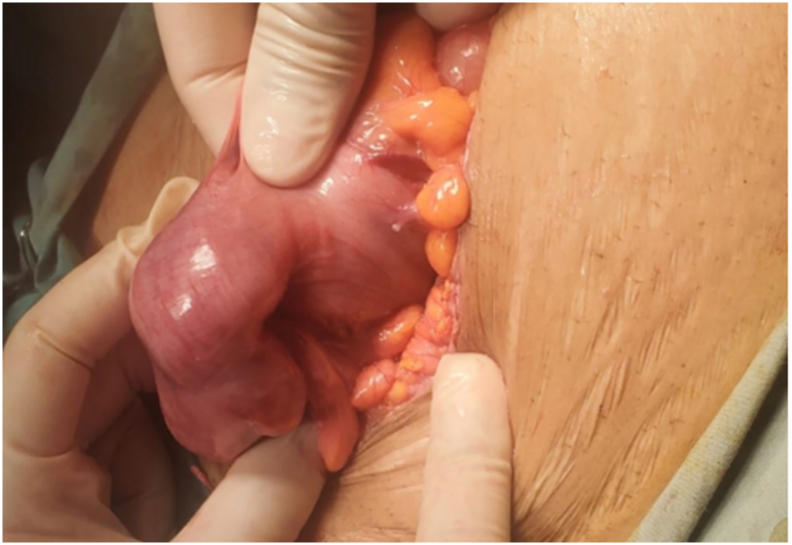
Intraoperative photograph. The image shows that the tumor within the sigmoid colon was mobilized for oncologic resection during lower midline laparotomy.

On December 22, 2024, the patient underwent surgical intervention via lower midline laparotomy. The open surgical approach was selected because of the large tumor size, uncertain resectability, and need for wide oncologic margins. Intraoperatively, a large, firm mass was identified in the sigmoid colon without evidence of peritoneal dissemination or distant metastases. The tumor was located in the sigmoid colon with no direct invasion of the adjacent structures. Careful assessment revealed that the mass did not involve the uterus, bladder, or other pelvic organs, allowing for complete mobilization and oncologic resection. Despite its large size, the tumor was well circumscribed, facilitating complete oncologic resection with 8-cm proximal and 5-cm distal margins, followed by primary colorectal anastomosis (Fig. 5). No complications occurred during this procedure.

**Fig. 6:**
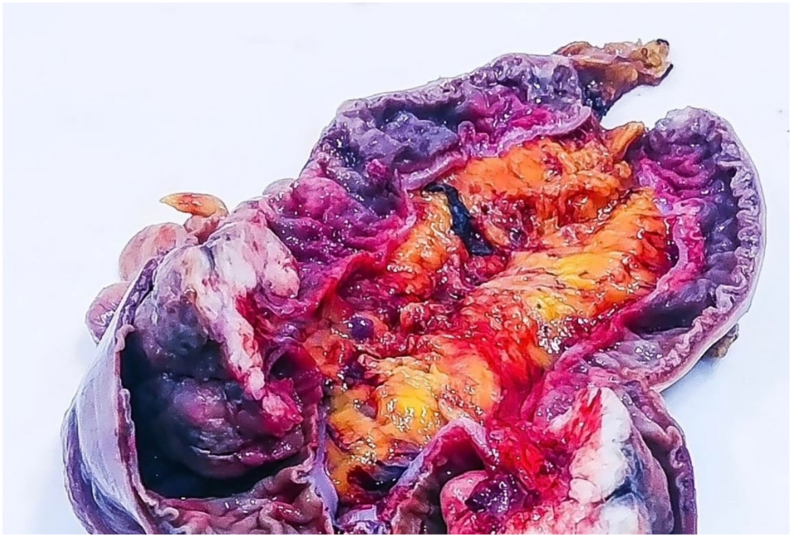
Gross surgical specimen. The resected segment of the sigmoid colon was cut open to display a large polypoid tumor mass. The final pathological examination confirmed a malignant peripheral nerve sheath tumor (MPNST) measuring approximately 6 cm in its largest dimension.

Final histopathological analysis confirmed the diagnosis of a malignant peripheral nerve sheath tumor (MPNST) with clear resection margins (R0 resection) (Fig. 6). The patient recovered well postoperatively and was discharged on the fifth postoperative day. At the 6-month follow-up in June 2025, the patient remained asymptomatic, with no evidence of local recurrence or distant metastases on clinical examination and imaging studies.

## 3 Discussion

This case represents one of fewer than ten documented instances of primary colonic MPNST, contributing valuable insights to an extraordinarily limited literature base. A comparative analysis of published cases (Table 3) revealed several critical patterns that inform our understanding of this rare entity [[6], [7], [8], [9], [10], [11], [12]].

**Table 3:** Literature comparison of colonic MPNST cases.

Author	Location	Patient age/sex	Tumor size (cm)	Association	Clinical presentation	Treatment	Outcome	Follow- up
Present Case (2025)	Sigmoid Colon	46 / F	6.0 × 5.3 × 5.2	Sporadic	Rectal bleeding, abdominal pain	Sigmoidectomy, R0 resection	Disease-free	6 months
[11]	Sigmoid Colon	55 / M	10 × 5	Sporadic	Abdominal pain, nausea, vomiting	Sigmoidectomy, stoma creation	Not specified	6 months
[3]	Transverse Colon	65 / M	20 × 18 × ll 10	Sporadic	Large abdominal mass, weight loss	Wide local excision, colostomy reversal	No residual disease	6 months
[6]	Splenic Flexure	32 / F	10 × 8	NF1	Dull abdominal pain, palpable lump	Segmental resection	Local recurrence	18 months
[7]	Descending Colon	60 / M	Large mass	Sporadic	Colicky abdominal pain	Left hemicolectomy, exploratory laparotomy	Recurrent MPNST	1 year
[9]	Ascending Colon	Newborn / M	5	Sporadic	Intestinal obstruction, vomiting	Right hemicolectomy	Disease-free	17 months
[23]	Small Bowel/Colon	71 / M	10 (primary)	Sporadic	Abdominal pain, anorexia, weight loss	Palliative debulking	Died of metastatic disease	<6 months

The literature comparison demonstrates a broad age distribution ranging from neonatal presentation to the seventh decade of life, with a slight male predominance (five males, three females). Our 46-year-old female patient fell within the middle-aged range of reported cases. The clinical presentations are uniformly non-specific, including abdominal pain (75 % of cases), gastrointestinal bleeding (25 %), and constitutional symptoms such as weight loss. This non-specificity contributes to diagnostic delays, as evidenced by our patient's four-year symptomatic period before the definitive diagnosis. Notably, only one case [6] was associated with NF1, in contrast to the 20–50 % association rate reported for MPNSTs [4]. This suggests that colonic MPNSTs may predominantly occur as sporadic tumors, although the small sample size limits the definitive conclusions.

The definitive diagnosis of colonic MPNST relies critically on comprehensive immunohistochemical analysis, as morphological features alone cannot distinguish MPNST from other spindle cell tumors. In our case, the diagnostic IHC panel served multiple crucial functions: (1) confirmation of neural crest origin through diffuse S-100 and SOX-10 positivity, (2) exclusion of GIST through negative CD117 and DOG-1 staining, and (3) exclusion of smooth muscle neoplasms through negative desmin and SMA staining [13].

The S-100 protein, while not entirely specific for MPNST, demonstrates positive staining in approximately 50–70 % of cases when combined with appropriate morphology. SOX-10, a more recently recognized marker, shows higher specificity for neural crest-derived tumors and has become increasingly valuable for MPNST diagnosis [15]. The negative staining pattern for GIST markers (CD117 and DOG-1) is particularly important given the higher prevalence of GISTs in the gastrointestinal tract and their morphological similarity to MPNST.

Analysis of the treatment outcomes revealed patterns of the aggressive nature of colonic MPNSTs. Among the cases with adequate follow-up data, the recurrence rate approached 40 % (3 of 8 cases), with two cases developing local recurrence and one progressing to metastatic disease, resulting in death within six months. This high recurrence rate aligns with the generally poor prognosis associated with MPNSTs, which have five-year survival rates of 35–60 % depending on various prognostic factors [16].

Complete surgical resection with clear margins (R0 resection) remains the cornerstone of treatment, and is the most critical prognostic factor for long-term survival. Our patient achieved R0 resection with wide margins (8 cm proximal and 5 cm distal), which may have contributed to the favorable short-term outcomes observed. However, the patient with the longest follow-up period (17 months) also achieved disease-free survival, suggesting that complete resection may provide durable control in selected cases.

The role of adjuvant radiation therapy and chemotherapy in colonic MPNSTs remains undefined owing to the extreme rarity of these tumors and lack of specific clinical trials. It is critical to distinguish the management of these sarcomas from that of conventional colon adenocarcinomas. While adjuvant chemotherapy is the standard of care for Stage III and high-risk Stage II colon adenocarcinoma, these recommendations are based on completely different tumor biology and do not apply to MPNSTs [17,18]. Similarly, adjuvant radiation is not routinely used for colon adenocarcinoma and has no established role in primary colonic MPNSTs [18].

Therefore, management decisions must be extrapolated from guidelines for soft tissue sarcomas. Generally, MPNSTs demonstrate relative resistance to conventional chemotherapy and radiation therapy, with response rates typically lower than those of other soft tissue sarcomas [19]. Adjuvant therapy is typically considered for high-grade tumors, positive resection margins, large tumors (>5 cm), and metastatic diseases.

In our case, adjuvant therapy was not administered given the achievement of complete R0 resection, the intermediate size of the tumor, and the lack of an established benefit for adjuvant treatment in this specific anatomical location and histological subtype. However, newer targeted therapies and immunotherapeutic approaches are under investigation for MPNSTs, particularly those with specific molecular signatures, and may represent future therapeutic avenues [20].

Given the aggressive nature and high recurrence potential demonstrated in the literature, intensive surveillance is paramount for patients with colonic MPNST, even after successful R0 resection. Our recommended surveillance protocol, which is in line with established guidelines for high-risk soft tissue sarcomas [21], includes clinical evaluation and cross-sectional imaging every six months for the first three years, followed by annual surveillance thereafter. This approach is based on the observation that most recurrences occur within the first 2–3 years after initial treatment.

The pattern of failure in reported cases includes both local recurrence and distant metastatic disease, necessitating comprehensive surveillance including chest imaging for pulmonary metastases, which represent the most common site of distant spread in MPNST [22].

The primary limitation of this report, similar to all published cases, is the relatively short follow-up period of six months. Given the aggressive nature of MPNSTs and the documented late recurrences in other cases, this timeframe is insufficient to draw definitive conclusions regarding long-term disease control. Extended follow-up of our patient and systematic reporting of additional cases are essential to better define the optimal management strategies. Additionally, the quality of some imaging findings may limit detailed radiological assessments, although key diagnostic features remain adequately demonstrated.

The extreme rarity of colonic MPNSTs limits their ability to conduct prospective studies and to establish evidence-based treatment guidelines. Future research should focus on the molecular characterization of these tumors to identify potential therapeutic targets and prognostic biomarkers that could guide treatment decisions.

## 4 Patient perspective

The patient expressed relief after receiving a definitive diagnosis after a prolonged four-year period of symptoms. The patient was satisfied with the outcome of the surgery and understood the rationale for long-term surveillance, to which she remained fully committed.

## 5 Conclusion

This report describes an exceptionally rare case of sporadic MPNST that originated in the sigmoid colon. A comparison with published cases revealed that colonic MPNSTs present significant diagnostic challenges owing to non-specific symptoms and morphological similarity to more common mesenchymal tumors. The high recurrence rate (approximately 40 %) reported in literature underscores the aggressive nature of these neoplasms and the critical importance of complete surgical resection with clear margins. Definitive diagnosis requires comprehensive immunohistochemical analysis, with S-100 and SOX-10 positivity confirming a neural crest origin, while negative staining for GIST and smooth muscle markers excluded more common differential diagnoses. Long-term surveillance is essential given the propensity for late recurrence and metastatic progression. This case contributes to the limited but growing body of literature on this rare entity and emphasizes the need for continued research to optimize the diagnostic and therapeutic approaches.

## Consent for publication

Written informed consent was obtained from the patient for the publication of this case report and any accompanying images and clinical details. A copy of the written consent form is available for review by the Editor-in-Chief of the Journal upon request.

## Ethical approval

**Institution Name**: Faculty of Medicine and Health Science, Sana'a University.

**Reason for Exemption**: In accordance with our institutional guidelines, formal ethics committee approval is not required for a retrospective case report detailing the clinical course of a single patient. The study was conducted in full compliance with the ethical principles of the Declaration of Helsinki, and written informed consent for publication was obtained directly from the patient.

## Guarantor

The guarantors of this work are the first author, Dr. Mohammed Mohammed Al-Shehari, and the corresponding author, Dr. Haitham M. Jowah. Both authors accept full responsibility for the integrity of the work as a whole, from inception to the published article, had access to all the data, and shared in the decision to submit for publication.

## Research registration number

Not applicable.

## Declaration of Generative AI and AI-assisted technologies in the writing process

During the preparation of this study, we used Google's large language model to improve the language, clarity, and overall structure of the manuscript. After using this tool, the authors reviewed and edited the content as needed, and took full responsibility for the content of the publication.

## Funding

No funding was received from any public, commercial, or not-for-profit agencies for the research, authorship, and/or publication of this article. Therefore, there were no study sponsors involved in the collection, analysis, or interpretation of data; in the writing of the manuscript; or in the decision to submit the manuscript for publication.

## Author contribution

• Conceptualization and Writing: H.M.J. conceptualized the case report and drafted the original manuscript.
• Surgical and Clinical Management: M.M.A. was the primary surgeon. K.S.G assisted in the surgical procedure, clinical management, and data collection.
• Radiological Analysis: M.A. and M.A.Q. performed and interpreted the radiological studies (CT and MRI) and contributed the imaging figures.
• Pathological Analysis: W.A.A. performed the histopathological and immunohistochemical examinations and provided the pathological interpretations.
• Review and Final Approval: All authors critically reviewed and edited the manuscript and approved the final version for publication.

## Conflict of interest statement

The authors declare that they have no conflicts of interest.

## Data Availability

All data generated or analyzed in this study are included in this published article. Further inquiries can be directed to the corresponding author.
